# Combined genome-scale fitness and paralog synthetic lethality screens with just 44k clones: the IN4MER CRISPR/Cas12a multiplex knockout platform

**DOI:** 10.1101/2023.01.03.522655

**Published:** 2023-01-03

**Authors:** Nazanin Esmaeili Anvar, Chenchu Lin, Lori L. Wilson, Annabel K. Sangree, Xingdi Ma, Medina Colic, John G. Doench, Traver Hart

**Affiliations:** 1– Department of Bioinformatics and Computational Biology, The University of Texas MD Anderson Cancer Center, Houston, TX, USA; 2– Graduate School of Biomedical Sciences, The University of Texas MD Anderson Cancer Center UTHealth, Houston, TX, USA; 3– Genetic Perturbation Platform, Broad Institute of MIT and Harvard, Cambridge, MA, USA; 4– Department of Cancer Biology, The University of Texas MD Anderson Cancer Center, Houston, TX, USA

## Abstract

Genetic interactions mediate the emergence of phenotype from genotype, but initial technologies for multiplex genetic perturbation in mammalian cells suffer from inefficiency and are challenging to scale. Recent focus on paralog synthetic lethality in cancer cells offers an opportunity to evaluate different CRISPR/Cas multiplexing technologies and improve on the state of the art. Here we report a meta-analysis of CRISPR genetic interactions screens, identifying a candidate set of background-independent paralog synthetic lethals, and find that the CRISPR/enCas12a platform provides superior sensitivity and assay replicability. We demonstrate that enCas12a can independently target up to four genes from a single guide array, and build on this knowledge by constructing a one-component library that expresses arrays of four guides per clone, a platform we call ‘in4mer’. Our genome-scale human library, with only 44k clones, is substantially smaller than a typical CRISPR/Cas9 monogenic library while also targeting more than two thousand paralog pairs, triples, and quads. Proof of concept screens in two cell lines demonstrate discrimination of core and context-dependent essential genes similar to that of state of the art CRISPR/Cas9 libraries, as well as detection of synthetic lethal and masking (also known as buffering) genetic interactions between paralogs of various family sizes, a capability not offered by any extant library. Importantly, the in4mer platform offers a fivefold reduction in the number of clones required to assay genetic interactions, dramatically improving the cost and effort required for these studies.

## Introduction

Pooled library CRISPR screens have revolutionized mammalian functional genomics and cancer target finding, with over a thousand screens conducted in cancer and other cell lines with CRISPR knockout libraries to identify background-specific genetic vulnerabilities^1,2^. However, despite initial efforts to assay genetic interactions^3–7^, CRISPR-mediated multiplex perturbations have proven complex and costly. Recently, focus has shifted to identifying synthetic lethal relationships between paralogs, homologous genes in a genome which share a common ancestor. Paralogs are an attractive target for genetic interaction studies because computational analyses have shown that they are enriched for synthetic lethals^8,9^ and because the mechanism of action of targeted chemotherapeutic agents often relies on inhibition of paralog gene products to mediate cell toxicity, leading to a disconnect between monogenic knockout screens for genetic vulnerability and for drug response in the same cells.

To this end, researchers have developed multiplex CRISPR perturbation methods to survey paralog synthetic lethals. Dual Cas9, orthogonal *S. pyogenes* and *S. aureus* Cas9, hybrid Cas9/Cas12a, and Cas12a-only approaches have been applied to knock out panels of paralog pairs in several cell lines^9–13^. However, with the application of different experimental and informatic pipelines, comparison across studies has been difficult.

Here we describe a meta-analysis of paralog genetic interaction screens in human cells, identifying a set of background-independent paralog synthetic lethals and demonstrate that the enhanced version of Cas12a from *Acidaminococcus sp*. (enAsCas12a, hereafter referred to as enCas12a) provides the best combination of sensitivity and simplicity for genetic interaction studies. Building on our prior work^9,14,15^, we show that the enCas12a system can reliably utilize guide expression arrays encoding at least four gRNA. Finally, we present the in4mer platform, a system for constructing libraries consisting of four-guide arrays that target specified sets of one to four genes independently. Our genome-scale human library based on the in4mer platform is, with 44,000 clones, approximately 30% smaller than standard whole-genome libraries. In addition, the same library targets more than two thousand paralog families of size two, three, and four, in a footprint fivefold smaller than comparable synthetic lethality libraries. This combination of features is not available with any other CRISPR perturbation platform.

## Results & Discussion

**Figure1.**
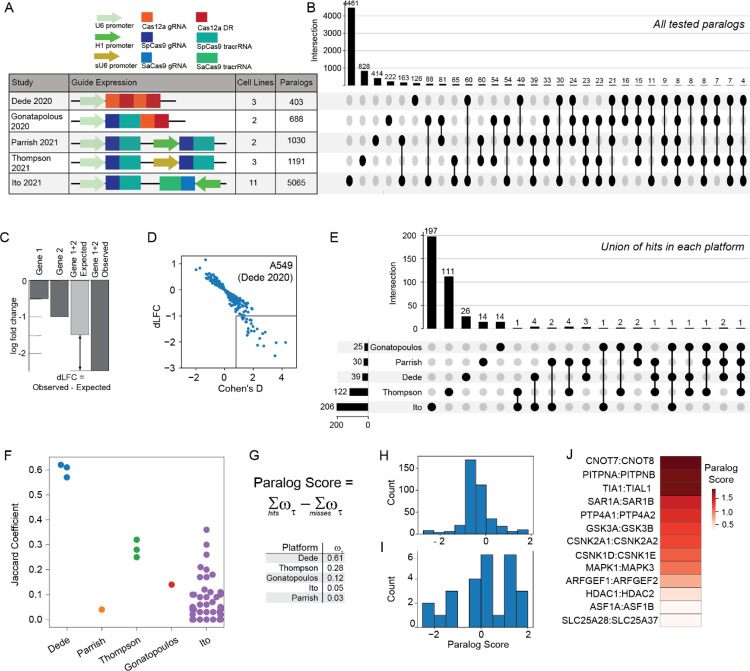
Comparative analysis of synthetic lethality screens. **A)** Different multiplex CRISPR perturbation methods applied to assay paralog synthetic lethality. B) Tested paralog pairs in each study. Upset plot shows the intersection of pairs across different studies. C) Quantifying synthetic lethality between paralog pairs. Single mutant fitness (SMF) is the mean log fold change of gRNAs that target an individual gene. Expected double mutant fitness (DMF) is calculated as the sum of SMF of gene 1 and gene 2. Delta Log Fold Change (dLFC) is the difference between observed and expected fold change and is used as a genetic interaction score. D) dLFC vs. Cohen’s D in one data set, A549 screen in Dede^9^. E) Comparison of union of hits across all cell lines in each study. F) Jaccard coefficient comparing within-study hits across all pairs of cell lines within each study. G) The “paralog score” is the weighted sum of hits minus the weighted sum of misses; i.e. where the gene pair is assayed but not a hit. Weights are the median of the platform-level Jaccard coefficients from (F). H). Histogram of paralog scores of 388 hits across all 5 studies. I) Histogram of paralog scores across 26 hits in >1 study. J) Thirteen candidate “paralog gold standards” with paralog score > 0.25 and hit in more than one study.

Technologies for interrogating mammalian genetic interactions are of high interest. Shortly after the adaptation of CRISPR technologies to genome-wide knockout screens, researchers began developing methods for multiplex knockouts^3–7^. With the discovery that paralogs are both systematically underrepresented in pooled library screens and likely offer the highest density of genetic interactions, recent focus has been on paralog synthetic lethality, with five independent studies each targeting hundreds of paralog pairs in multiple cell lines^9–13^. However, evaluating the quality and consistency of these studies has proven difficult, since each uses different technology and custom analytics pipeline for hit calling, and overlap between the targeted paralog pairs in each study is surprisingly slim (Figure 1A,B).

We developed a unified genetic interaction calling pipeline, based on measuring a pairwise gene knockout’s deviation from expected phenotype (delta log fold change, dLFC) and the effect size of this deviation (Cohen’s D) (Figure 1C,D). After performing background-specific normalization (see Methods), we classified paralogs as synthetic lethal if they met both dLFC and Cohen’s D thresholds. A total of 388 gene pairs were scored as hits across all five multiplex perturbation platforms (Figure 1E)

Using this pipeline, we found the large majority of paralog synthetic lethals to be platform-specific. To aid in comparing hits between and across pipelines, we developed a platform quality score that broadly measures how replicable these synthetic lethal technologies are in different cell lines. We reasoned that, like essential genes, most paralog synthetic lethals maintain their synthetic lethality across most or all cell lines and that relatively few will be context-specific. We therefore calculated the Jaccard coefficient of each pair of cell lines screened by a particular platform, and took the median of each platform’s Jaccard coefficients as the platform quality score (Figure 1F).

We then calculated a paralog confidence score for each gene pair by taking the sum of each hit, weighted by the platform quality score, and subtracting the sum of each experiment in which the pair was assayed but not deemed a hit, also weighted by quality score. Using this approach, paralog pairs that are hits in multiple high-quality screens outweigh pairs that are hits in screens with lower replicability or pairs that are background-specific hits in high-scoring screens. We further filtered for hits that are detected in more than one platform, minimizing the bias toward paralog pairs that are only assayed in one set of screens. We identified a total of 26 gene pairs that meet these criteria, which show a broad range of paralog scores, and we classify the top 13 hits (score >= 0.25) as “paralog gold standards” (Figure 1H–J).

### Optimizing the enCas12a system for multiplex perturbations

The Cas12a system enables multiplexing beyond two targets^12,16^. Based on the consistency of the enCas12a results in the paralog screens and its potential applications to higher-order multiplexing, we explored whether crRNA arrays longer than two guides could be utilized at scale. Guide RNA design is a critical factor in all CRISPR applications, and compared to >1,000 whole-genome screens in cancer cell lines performed with Cas9 libraries, empirical data on enCas12a guide efficacy is relatively sparse. We tested more than 1,000 crRNA from the CRISPick design tool targeting exons of known essential genes and found very strong concordance between the CRISPick on-target score and the fold change induced by the gRNA (Data not shown). We therefore considered CRISPick designs for all subsequent work.

**Figure 2.**
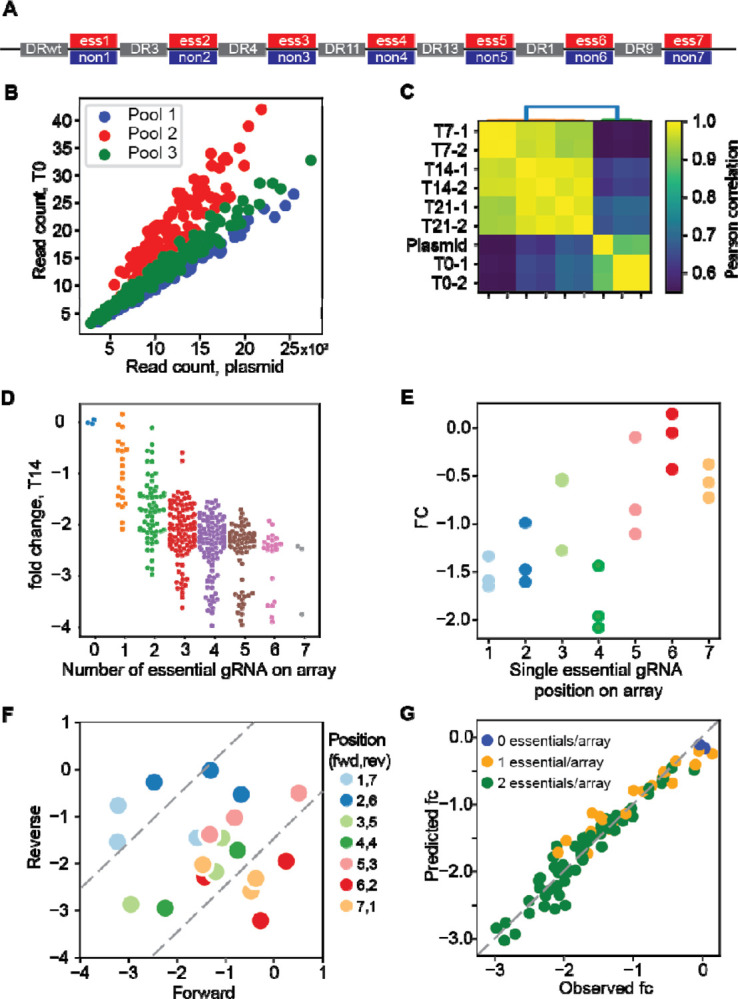
Multiplexing beyond 2 guides. (A) 7mer arrays were constructed with all combinations of either an essential or nonessential guide at each position (2^7=128 species), using the same DR sequences at each position, in three independent sets with unique gRNA sequences targeting the same genes at each position (n=384 total). (B) Guide sets were evenly represented in the combined pool before and after packaging and transduction (C) 7mer guide array representation is consistent across replicates and variation is consistent with high quality screens. (D) Fold change of guide arrays vs. number of essential guides on the array (n=384 arrays). (E) Fold change vs. position of essential guide on array, for all arrays encoding one essential guide (six nonessentials). (F) Fold change of guide arrays encoding one essential per array, forward vs. reverse orientation. Essential guides expressed at positions 6 and 7 deviate from the diagonal, indicating position-specific degradation of function. (G) A regression model trained on arrays encoding single essentials predicts the fold change of arrays targeting two essentials.

We have previously shown that arrays encoding two crRNA show no position effects^9,14,15^, but little is known about longer arrays. We constructed arrays of up to 7 gRNA to evaluate the maximum length that would yield gene knockout efficiency sufficient for pooled library negative selection screening. A set of seven essential and nonessential genes were selected and assigned to each array position 1–7. A single guide RNA was selected for each gene, and arrays were constructed such that at each position the essential or nonessential gRNA was randomly chosen, for a total library diversity of 128 array sequences (Figure 2A). The process was repeated twice, using different gRNA targeting the same genes, creating triplicate 128-clone pools targeting the same seven essential and nonessential genes in all combinations (Figure 2B).

We cloned the pools into the pRDA_550 vector (see Methods), a one-component lentiviral vector expressing the enCas12a CRISPR endonuclease and the *pac* resistance gene from an EIF1a promoter and the crRNA array from a human U6 promoter. After lentiviral packaging, we transduced K562 cells, a BCR-ABL leukemia cell line commonly used for functional genomics, with the library at 1000x coverage. After puromycin selection, samples were collected at 7, 14, and 21 days, and amplicons were sequenced to measure the relative abundance of 7mer arrays (Figure 2C). After normalization (see Methods), arrays with no essential gRNA showed no sign of negative selection compared to arrays with any number of essential gRNA. Arrays with multiple essential guides showed increasing loss of fitness, reaching a threshold at 4 to 5 essential guides per array (Figure 2D).

To evaluate position-level effects, we considered arrays encoding a single essential gRNA at any of the seven positions. Across the three replicates, we consistently observed greater fold change at the first four positions compared to the last three positions on the array (Figure 2E). We further tested whether this efficiency drop at the end of the array was a position-dependent effect or the result of unfortunate guide or gene selection. We constructed a reversed array with the same gRNA targeting the same genes in reverse order (one essential gRNA per array) and re-screened the same cells. When comparing the fold change of the forward array with the reverse array, observed fold changes on the diagonal indicate gene- and guide-level effects independent of position, while deviations from the diagonal indicate position-specific effects. Our data confirm that the first four to five gRNA show no position-specific effects, but positions six and seven show marked deviation from the diagonal (Figure 2F). Based on these observations, we conservatively conclude that the enCas12a system using the pRDA_550 vector can effectively express and utilize arrays of four gRNA.

We also evaluated whether the 7mer array could be used to identify combinatorial phenotypes. We trained a linear regression model using a binary encoding of guide arrays as a predictor (where nonessential = 0 and essential = 1) and observed fold change as a response variable (see Methods). The regression model provides excellent prediction of fold change for arrays encoding two essentials (R^2 = 0.78–0.91 for the three pools) from the sum of calculated single-guide position-level regression coefficients (Figure 2G). A similar approach predicted with high accuracy the fold change of arrays with three essentials, except where the model predicts fold changes beyond the dynamic range of our assay. These observations are consistent with the multiplicative model of independent phenotypes, which predicts that the result of independent loss of fitness perturbations is the sum (in log space) of the fold changes of the individual fitness perturbations. It further supports the utility of the enCas12a platform for multiplex perturbation and detection of genetic interactions, which are simply deviations from the expected phenotype according to this model, because the null model accurately fits the data for independent combinatorial perturbations.

### The in4mer platform for single and combinatorial perturbation

**Figure 3.**
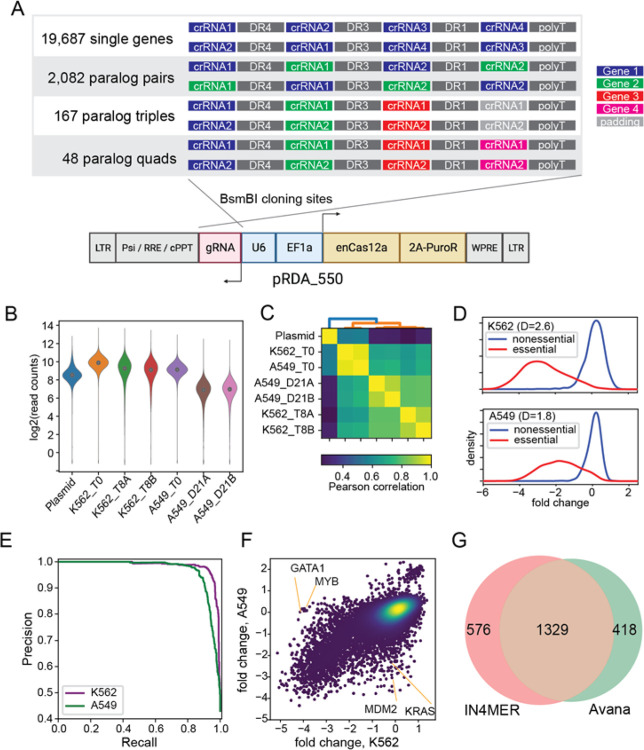
In4mer platform for whole-genome screening. (A) In4mer human whole-genome library targets single genes and paralog pairs, triples, and quads with arrays of 4 enCas12a gRNA. Each gene or gene family is targeted by two arrays encoding the same gRNA in different order. Commercially synthesized oligo pools are cloned into the one component pRDA_550 lentiviral vector; schematic created in Biorender. (B-F) Screening in K562 CML cells and A459 lung cancer cells. (B) Read counts from the plasmid and experimental timepoints after lentiviral transduction. (C) Correlation of sample read counts. T0 samples and endpoint replicates are highly correlated. (D) Fold change distributions of arrays targeting reference essential (red) and nonessential genes (blue) in K562 and A549. D, Cohen’s D statistic. (E) Precision/recall analysis from ranked mean fold change of arrays targeting each gene, calculated against reference essential and nonessential genes. (F) Mean fold change of arrays targeting each gene/gene family in K562 vs A549 shows high consistency (Pearson’s r=0.74) as well as background-specific essential and tumor suppressor genes. (G) Comparison of K562 hits from in4mer and DepMap data.

With confidence that the enCas12a platform supports independent utilization of four to five guides expressed from a single array, we designed a genome-scale library that targets both protein-coding genes and paralog families in the same pool. Each array encodes four distinct gRNA, each with its own DR sequence selected from the top performers in De Weirdt *et al*^14^. (Figure 3A). The library targets each of 19,687 protein coding genes with one four-guide array encoding 20mer crRNA sequences from the top four guide selections from the CRISPick algorithm, and a second four-guide array using the same guides in a different orientation. The library also targets 2,082 paralog pairs with a single array encoding two gRNA per gene and a second array encoding the same gRNA in a different orientation (see Methods for paralog selection strategy). Additionally, 167 paralog triples and 48 paralog quads are targeted by two arrays, with each array encoding a single guide targeting each gene (arrays targeting triples are padded with a fourth guide targeting a randomly selected nonessential gene). For triples and quads, the arrays encode different gRNA sequences (Figure 3A). Total library size is 43,972 4mer CRISPR arrays, including 4 arrays with 4 guides each targeting EGFP. Since the leading direct repeat sequence is already on the pRDA_550 backbone, the library can be commercially synthesized as a 212mer oligo pool.

We conducted screens in K562, a *BCR*-*ABL* chronic myeloid leukemia cell line, and in A549, a *KRAS* lung cancer cell line with wildtype *TP53*, using standard CRISPR screening protocols (500x library coverage, 8–10 doublings). Array amplicons were sequenced using single-end 150-base sequencing on an Illumina NextSeq 500. Quality control metrics met expectations (Figure 3B), and the abundance distributions of T0 reference samples and endpoint replicates were highly correlated (Figure 3C). Fold changes calculated relative to T0 showed increasing correlation when comparing clones targeting the same gene/gene family within one replicate (n=22k targets, r=0.78), all clones between two technical replicates derived from the same transduction (n=44k arrays, r=0.86), and the mean of clones targeting the same gene across technical replicates (n=22k targets, r=0.92). In4mer guides effectively discriminated reference essential genes from nonessentials (Figure 3D), with Cohen’s D statistics consistent with high-quality screens in DepMap (DepMap median Cohen’s D = 2.7), and yielding precision-recall curves comparable to effective CRISPR/Cas9 whole genome screens (Figure 3E). Comparison of K562 and A549 shows expected background-specific essential genes such as *KRAS* and *MDM2* in A549 and lineage-specific transcription factors *MYB* and *GATA1* in K562 (Figure 3F), and essential genes in the in4mer K562 screen are highly concordant with prior K562 pooled library CRISPR knockout fitness screens (Figure 3G).

**Figure 4.**
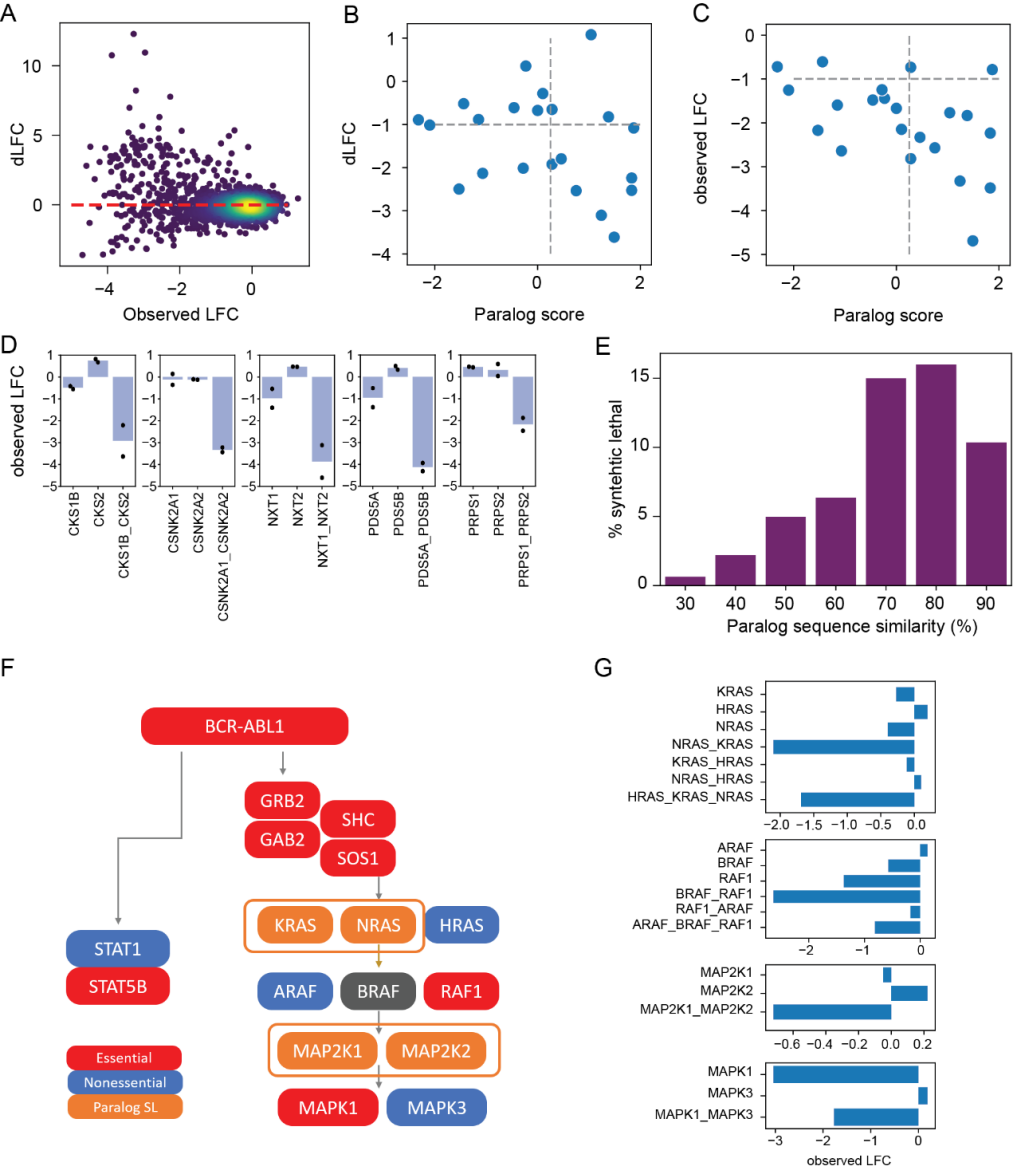
Synthetic lethality in K562. (A) Fold change vs. dLFC for >2,000 paralog families. (B) dLFC vs. Paralog Score from meta analysis of published paralog screens. Of 11 paralogs with score > 0.25, 8 show dLFC < −1 in K562. C) Fold change vs. paralog score. Most paralogs are essential, regardless of synthetic lethality. D) Selected synthetic lethals showing fold change of single and double knockout. Bar chart, mean fold change. Scatter plot, fold change of single array of gRNA (mean of 2 replicates). E) Fraction of synthetic lethal paralogs by amino acid sequence similarity. F) Pathway activation by BCR-ABL1 fusion in K562 cells. Red, essential gene in in4mer screen; blue, nonessential; orange, synthetic lethal paralog pair. G) Single, double, and triple knockout fold change of paralogs in MAP kinase pathway as shown in F).

To evaluate paralog genetic interactions, we used the multiplicative model to calculate expected phenotype (i.e, log fold change) of multiplex knockouts by adding the log fold change of the single gene knockouts. We then compared the observed mean fold change of guide arrays targeting the 2,082 gene pairs and 215 families of size 3 or 4 with the expected fold change under the multiplicative null model to calculate a delta log fold change (dLFC) that represents the magnitude of the genetic interaction (Figure 4A). Gene pairs with strongly negative dLFC are highly concordant with the gold standard paralog synthetic lethals described above. The in4mer library targets 22 of the 26 gene pairs that are hits in >1 of the previously published screens, and 11 of the 13 with paralog scores > 0.25. Of those 11, 8 have dLFC < −1 in the K562 screen, for an estimated sensitivity of 73% (Figure 4B). Moreover, 9 of 11 pairs (81%) with high paralog score are essential, regardless of synthetic lethality, as are 9 of 11 pairs (81%) with lower paralog scores (Figure 4C), consistent with either one paralog being essential or synthetic lethality below our strict threshold of dLFC < −1. Other paralogs show strong genetic interaction relative to their null expectation (Figure 4D), with a strong relationship between sequence identity and probability of genetic interaction (Figure 4E), in keeping with prior observations by De Kegel & Ryan^8^.

An advantage of using well-characterized models like K562 is that it facilitates precise comparison with known biology. The *BCR-ABL* fusion oncogene in K562 activates the STAT and MAP kinase pathways, and we classify *ABL1*, *STAT5B*, and the *GRB2/SOS1/GAB2/SHC* signal transduction module as individual essentials (Figure 4F). None of the *ras* genes are individually essential, but *KRAS-NRAS* shows a strong synthetic lethality. Neither *KRAS-HRAS* nor *HRAS-NRAS* paralogs show genetic interaction, but the three-way *HRAS-KRAS-NRAS* clones also show strong essentiality, almost certainly due to the *KRAS-NRAS* interaction (Figure 4G). While it is known that RTK/MAP kinase signal transduction must flow through the *ras* genes, to our knowledge, this is the first time that *KRAS-NRAS* functional buffering has been demonstrated experimentally.

Beyond the *ras* genes, the rest of the MAP kinase pathway also shows the expected gene essentiality profile. *RAF1* is strongly essential, and while *BRAF* is slightly below our hit threshold, the *BRAF*-*RAF1* pairwise knockout shows evidence of independent additive phenotype. The third member of the paralog family, *ARAF*, is nonessential singly or in combination with the other *raf* paralogs and has not been shown to operate in this pathway. The MEK kinases, *MAP2K1*/*MAP2K2*, show greater fold change from pairwise loss than from either individually, though below our strict threshold for synthetic lethality. The ERK kinases, *MAPK1*/*MAPK3*, show strong preferential reliance on *MAPK1*. This is consistent with DepMap data for K562, which, unique among CML cell lines, shows this isoform-specific dependence, which is more commonly associated with melanoma cell lines with oncogenic *BRAF*. The MAP kinase pathway in A549 cells shows similar expected activation, with *KRAS* being strongly essential and *MAPK1-MAPK3* showing strong synthetic lethality (Figure 4G).

**Figure 5.**
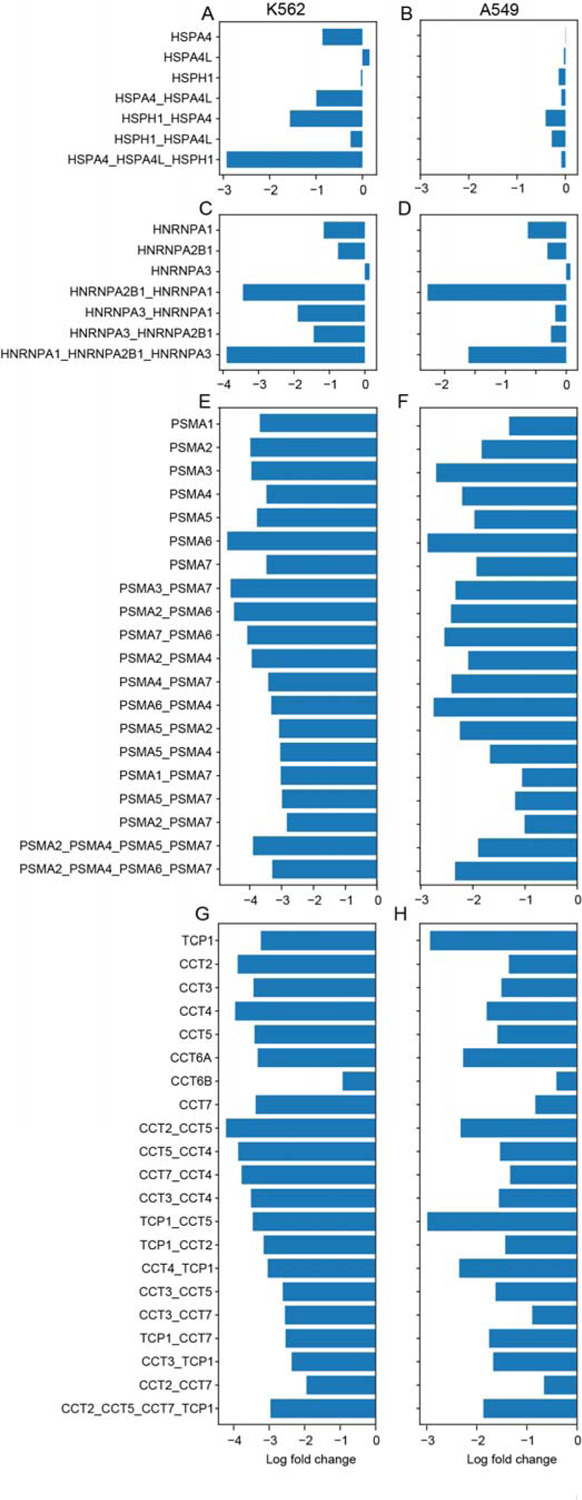
Higher order interactions. HSPA4 family shows triplex interaction in K562 (A) but not A549 (B). (C,D) Triplex fitness phenotype is often explained by duplex knockout, as in HNRNPA1 family example shown here. (E,F) Masking interactions in proteasome subunits. Knockout of two or more genes in the same essential complex yields no marginal fitness gain or loss compared to single gene knockout. (G,H) Masking interactions in CCT complex subunits.

We explored the 215 paralog triples and quads for evidence of higher-order genetic interactions. As noted above, the three-way interaction between *KRAS, NRAS*, and *HRAS* is explained by the *KRAS-NRAS* interaction. However, we did observe an intriguing interaction between *HSPA4* family of Hsp70-related chaperones. *HSPA4* shows moderate phenotype when knocked out singly or in a pair with either family member, and severe phenotype when all three are targeted (Figure 5A). Interestingly, this phenotype appears to be context specific, as it is absent in A549 cells (Figure 5B). Platform sensitivity to three-way interactions seems comparable to that of two-way interactions, despite each construct only expressing a single guide targeting each gene, since three-way combinations that include observed pairwise synthetic lethals tend to show similar fold changes (for example, the *HNRNPA1* family, Figure 5C–D). The *HSPA4* family is the only three-way synthetic lethal we observe in our limited data.

Higher-order masking interactions, on the other hand, are strikingly visible. Both the core proteasome and the Chaperonin-Containing TCP1 (CCT) complex are comprised of several weakly related proteins, which we target with three four-way constructs and numerous two-way constructs. Since both the proteasome and the CCT complex are universally essential to proliferating cells, knockdown of single subunits induces a severe fitness phenotype. Knockout of these genes in pairs or quads yields no additional phenotype, resulting in masking/positive genetic interactions in both K562 and A549 cells (Figure 5E–H).

## Conclusions

Confidence in any technology’s accuracy in assaying the existence, abundance, or activity of a biomolecule is tied to the method’s ability to recapitulate known biology. Absence of established gold standards arguably contributed to the shortcomings of RNAi based studies of mammalian gene function 17. We 18,19 and others 2 have created widely used reference sets of essential and nonessential genes for use in quality control of CRISPR and other knockdown/knockout loss of fitness screens. As CRISPR perturbation technology has advanced into genetic interactions, it has become clear that a similar gold standard for synthetic lethals would be highly useful 20.

Our meta-analysis of published screens for paralog synthetic lethals in human cells shows wide divergence in the paralogs assayed by each study and the repeatability of each screen, as measured by the Jaccard coefficient of hits in different cell lines. We reasoned that paralogs that showed synthetic lethality within and across screening platforms are likely to be globally synthetic lethal, analogous to core essential genes, and the fact that 12 of our 13 candidate reference paralogs show more than 70% identity -- and all are constitutively expressed -- is consistent with this interpretation.

Notably, the enCas12a platform from Dede et al 9 performed markedly better in terms of replicability. Based on this and our prior work with the CRISPR/Cas12a system 14,15, we tested the limits of the enCas12a system expressing guide arrays from the Pol III U6 promoter in a custom one-component lentiviral vector, pRDA_550. For longer arrays of 7 independent gRNA, we observed that position-specific loss of knockout efficiency did not arise until after the fourth or fifth gRNA in the array.

With confidence that the one-component CRISPR/enCas12a system efficiently targeted genes with long arrays, we designed a genome-scale library based on a standardized four-guide array. This platform, which we call in4mer, offers several advantages over the current state of the art. By targeting single genes with four independent gRNA, we lower the odds of any single guide fails to induce the desired phenotype, extending the work shown in De Weirdt et al 14. This reduces the number of reagents required to target each gene. In our prototype library only two reagents, each encoding the same four gRNA in different order, target each gene, yielding a genome-scale knockout library in only ~40,000 reagents, on par with Humagne and the Garnett lab Cas9 mini-library. The major advantage that the enCas12a system offers over the Cas9 equivalents is that the library is constructed from a single 212mer oligo pool and both cloning and amplicon sequencing are performed using essentially the same protocols as single-guide Cas9 screening, albeit with longer sequencing reads.

Building on this advantage, we include in the same library a set of reagents targeting more than 2,000 selected paralog pairs, triples, and quads. The 4mer arrays encode two guides targeting each of the two genes in a pair or one guide per gene in a triple or quad family. Prototype screens show very high sensitivity for synthetic lethality, detecting 8 of 11 (73%) of our reference paralogs. We show novel synthetic lethality between KRAS and NRAS in BCR-ABL fusion K562 cells, as well as dependency at the single or gene pair level at every node of the MAP kinase signal transduction pathway, recapitulating known biology but not, to our knowledge, shown in a single experiment before. We additionally discover a three-way synthetic lethality among members of the HSPA4 family of Hsp70-related genes. None of these genes shows LOF phenotype in A549 cells, suggesting a background-specific effect.

The human genome library constructed from the in4mer platform offers a miniaturized version of a genome-scale library, consistent with the Humagne 14, HD 21, and MiniLibCas9 22 libraries, coupled with a significant increase in the efficiency of genetic interaction platforms. The five paralog synthetic lethal studies used at least thirty constructs per gene pair tested, while the in4mer platform requires only six: two targeting each gene and two targeting the gene pair. This fivefold decrease in reagent requirements has major implications for the cost effectiveness in genetic interaction assays in mammalian cells, where the number of gene pairs and the diversity of cell/tumor lineages yield a vast search space.

## Methods

### Data preprocessing

To reanalyze the data from the 5 paralog screens, raw read counts were downloaded, and the same pipeline was applied to all of them. The following analysis was executed in Python notebooks and will be available at the Hart Lab github repository, though not currently available for this early version preprint. A pseudocount of 5 reads was added to each construct in each replicate, and read counts were normalized to 500 reads per construct. Log fold change (LFC) for each guide at late time point was calculated relative to the plasmid sequence counts.

The data from each study (except Thompson) was divided into three groups; the constructs that target single genes paired with non-essential/non_targeting gRNAs (N) in the first position (gene_N), in the second position (N_gene) and constructs that target gene pairs (A_B). LFC values of each group were scaled individually so that the mode of each group was set to zero. Next, all three groups were merged in one table. Before dividing Ito’s dataset into three groups, LFC values were scaled such that the mode of negative controls (non-essential_AAVS1) would be zero and also TRIM family was removed from this dataset to avoid false paralog pair discovery^13^. Since in Thompson’s study there was just one position for singleton constructs, LFC values were scaled so that the mode of negative controls (non-essential_Fluc) was set to zero. In the next step, LFC of each construct was calculated by the mean of LFC across different replicates.

### Calculating genetic interaction

For each gene, single gene mutant fitness (SMF) was calculated as the mean construct log fold change of gene-control constructs. The control was either non-essential genes or non-targeting gRNAs. For each gene pair, expected double mutant fitness (DMF) of genes 1 and 2 was calculated as sum of SMF of gene 1 and SMF of gene 2. The difference between expected DMF and the observed DMF, the mean LFC of all constructs targeting genes 1 and 2, was called dLFC.

Next step was calculating a modified Cohen’s D between observed and expected distribution of LFC of gRNAs targeting genes. Expected distribution of gRNAs targeting a gene pair, was calculated using expected mean and expected standard deviation.

expected_mean =μ1+μ2

$$\text{expected\_std }=\sqrt{{\left(\text{std}1\right)}^{2}+{\left(\text{std}2\right)}^{2}}$$

$$S_{\text{pooled }}=\frac{\sqrt{{\left(\text{ expected\_std }\right)}^{2}+{\left(\text{ observed\_std }\right)}^{2}}}{2}$$

$$\text{Cohen′sD}=\frac{\text{ expected\_mean }-\text{ observed\_mean }}{S_{\text{pooled }}}$$

Where:

- μ1 = The mean construct LFC of gene1 constructs
- μ2 = The mean construct LFC of gene2 constructs
- std1 = Standard deviation of LFC of gene1 constructs
- std2 = Standard deviation of LFC of gene1 constructs

In each cell line, the paralog pairs with dLFC less than −1 and Cohen’s D more than .8 were selected as hits. Cohen’s D more than .8 indicates large effect size between two groups, meaning that our expected and observed distribution of gRNAs are meaningfully separated. In total 388 paralog pairs were identified as hits across all the studies.

To identify the most consistent method in terms of hit identification, the Jaccard similarity coefficient of every pair of cell lines in each study was calculated by taking the ratio of intersection of hits over union of hits. For the studies that screened more than two cell lines, the final Jaccard score was the median of the calculated Jaccard score of all pairs of cell lines.


$$J(A,B)=\frac{\left|A\cap B\right|}{\left|A\cup B\right|}=\frac{\left|A\cap B\right|}{\left|A\right|+\left|B\right|-\left|A\cap B\right|}$$

### Scoring Paralog Pairs

Each hit was scored based on the cell lines in which it was identified as a hit; cell lines were weighted based on the Jaccard score of each study. We defined the “paralog score” as the sum of Jaccard scores of cell lines in which the paralog pair was identified as a hit minus the sum of Jaccard scores of cell lines in which the paralog pair was assayed but not identified as a hit (a “miss”). The distribution of scores is shown in Figure 1. The paralog pairs that scored more than 0.25 and were identified as a hit in two or more studies were listed as a reference set.


$$\text{ Paralog Score }={\text{Σ}}^{\text{hits}}\left(w_{n}\right)-{\text{Σ}}^{\text{misses }}\left(w_{n}\right)$$

### One-component CRISPR/enCas12a vector

To construct an all-in-one vector for expression of both Cas12a and a guide array, we first swapped in puromycin resistance in place of blasticidin resistance from pRDA_174 (Addgene 136476). We then tested four locations for the insertion of a U6-guide expression cassette; notably this cassette needs to be oriented in the opposite direction of the primary lentiviral transcript to prevent Cas12a-mediated processing during viral packaging in 293T cells. The construct with the best-performing location, between the cPPT and the EF1a promoter, was designed pRDA_550 (Addgene # pending). Synthesis of DNA and custom cloning was performed by Genscript.

### Library production

An oligonucleotide pool consisting of 43,972 four-plex guide arrays targeting 19,687 single genes, 2,082 paralog pairs, 167 paralog triples, and 48 paralog quads was synthesized by Twist Bioscience using the following template:

5’*AATGATACGGCGACCACCGA***cgtctcgAGAT**nnnnnnnnnnnnnnnnnnnnTAATTTCTACTATTGTAGATnnnnnnnnnnnnnnnnnnnnAAATTTCTACTCTAGTAGATnnnnnnnnnnnnnnnnnnnnTAATTTCTACTGTCGTAGATnnnnnnnnnnnnnnnnnnnnTTTTTT**GAATggagacg***ATCTCGTATGCCGTC TTCTGCTTG*-3’

This 212nt fragment contained engineered variant direct repeats (underlined) separating four 20nt guide sequences (lowercase ‘n’) flanked by BsmBI restriction sites (bold lowercase) with overhangs designed to matching the pRDA-550 vector (bold uppercase). Flanking sequences (italic) enabled PCR amplification of the oligo pool.

The pool of guide arrays was PCR amplified using KAPA HiFi 2X HotStart ReadyMix (Roche) using 20ng of starting template per 25ul reaction using primers CL_Amp_F (5’AATGATACGGCGACCACCGA-3’) and CL_Amp_R (5’CAAGCAGAAGACGGCATACGAGT-3’) at a final concentration of 0.3uM and the following conditions: denaturation at 95°C for 3 min, followed by 12 cycles of 20 s at 98°C, 30 s at 60°C, and 30 s at 72°C using a ramp rate of 2°C/s, followed by a final extension of 1 min at 72°C.

Full length amplicon (212nt) was purified using the Monarch PCR & DNA Cleanup Kit (New England Biolabs).

Three oligonucleotide pools were synthesized by Intergrated DNA Technologies and used to assembly three sets of 7mer library.

Both 4mer and 7mer purified amplicons were cloned into the pRDA-550 vector by BsmBI-v2 Golden Gate Assembly (New England Biolabs following the manufacturer’s instructions. A 2:1 (insert:vector) molar ratio was used in Golden Gate Assembly.

The final ligation products were desalted prior to transformation in bacterial cells using the Momarch PCR & DNA Cleanup Kit (New England Biolabs).

Ligation products were transformed into Endura Electrocompetent cells (Lucigen) and allowed to recover for 1 hour at 30°C. Transformed bacteria were diluted 1:100 in 2xYT medium containing 100ug ml^−1^ carbenicillin (Sigma) and grown at 30°C for 16 hours.

Transfection grade plasmid library was purified using the PureLink^™^ Plasmid Purification Kit (Invitrogen) and guide arrays were sequenced to confirm uniform and complete library representation.

### Cell culture

K562 and A549 cells were a gift from Tim Heffernan. Cell line identities were confirmed by STR fingerprinting by M.D. Anderson Cancer Center’s Cytogenetic and Cell Authentication Core (Promega Powerplex 16 High Sensitivity Assay). All cell lines were routinely tested for mycoplasma contamination using cells cultured in non-antibiotic medium (PlasmoTest Mycoplasma Detection Assay, InvivoGen).

All cell lines were grown at 37°C in humidified incubators at 5.0% CO_2_ and passaged to maintain exponential growth.

K562 cells were cultured in HEPES modified RPMI-1640 Medium (Sigma R5886) supplemented with 10% FBS (Sigma), 1mM sodium pyruvate (Gibco), 2mM L-alanyl-L-glutamine dipeptide (Gibco), 1X penicillin-streptomycin (Gibco), and 100 ug mL^−1^ Normocin (InvivoGen).

A549 cells were cultured in HEPES modified Dulbecco’s Modified Eagles Medium (Sigma D6171) supplemented with 10% FBS (Sigma), 1mM sodium pyruvate (Gibco), 2mM L-alanyl-Lglutamine dipeptide (Gibco), 1X penicillin-streptomycin (Gibco), and 100 ug mL^−1^ Normocin (InvivoGen).

### enCas12a Screens

Lentivirus was produced by the University of Michigan Vector Core. Virus stocks were not titered in advance. Transduction of the cells was performed at 1X concentration of virus using 8ug ml^−1^ polybrene transfection reagent (EMD Millipore).

Non-transduced cells were eliminated via selection with 2ug mL^−1^ puromycin dihydrochloride (Gibco). Selection was maintained until all non-transduced control cells reached 0% viability. Once selection with puromycin was complete, surviving cells were pooled and 500x coverage cells were harvested for a T0 sample (i.e. 500 cells per guide array). After T0, cells were harvested at 500X coverage at 8 (7 for 7mer), 14, and 21 days.

### Sequencing

Genomic DNA (gDNA) purification was achieved using the Mag-Bind Blood and Tissue DNA HDQ Kit (Omega Biotk) using magnetic bead compatible reagents. Purified gDNA was eluted in 10mM Tris-HCl pH 8.0, 1mM EDTA and quantified by fluorimetry using the Qubit dsDNA Broad Range Kit (ThermoFisher).

Illumina-compatible guide array amplicons were generated by amplification of the gDNA in a one-step PCR. Indexed PCR primers were synthesized by Integrated DNA Technologies using the standard 8nt indexes from Illumina (D501-D508 and D701-D712) as follows:

#### Dual-lib Forward Primer:

5’**AATGATACGGCGACCACCGAGATCTACAC**nnnnnnnnACACTCTTTCCCTACACGACGCT CTTCCGATCT*CTTGTGGAAAGGACGAAACACCG*-3’ (**i5 flow cell adapter** – i5 index – i5 read1 primer binding site – *Amplicon annealing sequence*)

#### Dual-lib Reverse Primer:

5’**CAAGCAGAAGACGGCATACGAGAT**nnnnnnnnGTGACTGGAGTTCAGACGTGTGCTCTTC CGATCT*ACCGACTCGGTGCCACTTTTTCAAGACCAG*-3’ (**i7 flow cell adapter** – i7 index – i7 read2 primer binding site – *Amplicon annealing sequence*)

Guide arrays were amplified from at least ~200X coverage DNA per replicate across multiple reactions, not exceeding 2.5ug gDNA per 50 ul PCR reaction. Each 50 ul reaction contained 1ul of each primer(10 uM), 1ul 50X dNTPs, 5% DMSO, 5 ul of 10X Titanium Taq Buffer, and 1ul of 10X Titanium Taq DNA Polymerase (Takara). Conditions for the PCR reactions were as follows: denaturation at 95°C for 60 s, followed by 25 cycles of 30 s at 95°C and 1 min at 68°C, followed by a final extension at 68°C for 3 min.

The indexed amplicons (360bp for 4mer, 501bp for 7mer) were purified by size selection using the E-Gel SureSelect II, 2% agarose (ThermoFisher). Purified amplicons were quantified by Qubit dsDNA High Sensitivity Assay Kit (ThermoFisher). Quality of the purified amplicons was determined using the D1000 ScreenTape Assay for TapeStation (Agilent). Purified amplicons were then pooled and sequencing was performed by NextSeq 500 sequencing platform (Illumina) with custom primers:

Forward: 5’-CTTGTGGAAAGGACGAAACACCGGTAATTTCTACTCTTGTAGAT-3’

Reverse: 5’-ACCGACTCGGTGCCACTTTTTCAAGACCAG-3’ (7mer only).

The 4mer library was sequence by read format of 151-8-8, single-end. The 7mer library was by read format of 151-8-8-151, paired-end.

### Screen data analysis

In4mer library sequencing reads were mapped to the library using only perfect matches. BAGEL2 was used to normalize sample level read counts and to calculate fold changes relative to the T0 reference using the BAGEL2.py *fc* option with default parameters^23^. Essential and nonessential genes were defined using the Hart reference sets from^18,19^. Since the library targets both individual genes and specific gene sets (paralogs), we calculated the average gene/geneset (hereafter ‘gene’) log fold change as the mean of the clone-level fold changes across two replicates. All fold changes are calculated in log2 space. Cohen’s D statistics were calculated in Python. Data for recall-precision curves were calculated using BAGEL2. We set an arbitrary threshold of fc < −1 for essential genes.

For genetic interaction analysis, the expected fold change was calculated as the sum of the gene-level fold changes for each individual gene in the geneset. Expected fc was subtracted from observed fc to calculate delta log fold change, dLFC, where negative dLFC indicates synthetic/synergistic interactions with more severe negative phenotype, and positive dLFC indicates positive/suppressor/masking interactions with less severe negative or more positive phenotype than expected. We set an arbitrary threshold of dLFC < −1 for synthetic lethality, and > +1 for masking/suppressor interactions.
